# Ectopic Expression of Hematopoietic SHIP1 in Human Colorectal Cancer

**DOI:** 10.3390/biomedicines8070215

**Published:** 2020-07-15

**Authors:** Matthias Schaks, Kristina Allgoewer, Nina Nelson, Patrick Ehm, Asmus Heumann, Florian Ewald, Udo Schumacher, Ronald Simon, Guido Sauter, Manfred Jücker

**Affiliations:** 1 Institute of Biochemistry and Signal Transduction, University Medical Center Hamburg-Eppendorf, 20251 Hamburg, Germany; matthias.schaks@helmholtz-hzi.de (M.S.); k.allgoewer@gmail.com (K.A.); fcsx044@studium.uni-hamburg.de (N.N.); p.ehm@uke.de (P.E.); 2 Department of Pathology, University Medical Center Hamburg-Eppendorf, 20251 Hamburg, Germany; aheumann@uke.de (A.H.); r.simon@uke.uni-hamburg.de (R.S.); g.sauter@uke.de (G.S.); 3 Department of General-, Visceral- and Thoracic-Surgery, University Medical Center Hamburg-Eppendorf, 20251 Hamburg, Germany; f.ewald@uke.de; 4 Department of Anatomy and Experimental Morphology, University Medical Center Hamburg-Eppendorf, 20251 Hamburg, Germany; uschumac@uke.de

**Keywords:** SHIP1, inositol 5-phosphatase, microsatellite instability, colorectal cancer, carcinoma

## Abstract

Colorectal cancer (CRC) is a heterogeneous disease that results from the accumulation of mutations in colonic mucosa cells. A subclass of CRC is characterized by microsatellite instability, which is thought to occur mainly through inactivation of the DNA mismatch repair genes *MLH1* and *MSH2*. The inositol 5-phosphatase SHIP1 is expressed predominantly in hematopoietic cells. In this study, the expression of SHIP1 in carcinomas and its putative correlation with clinicopathologic parameters, expression of DNA repair genes and microsatellite instability was investigated. By analyzing a multi-tumor tissue microarray, expression of SHIP1 was detected in 48 out of 72 cancer entities analyzed. The expression of SHIP1 protein of 145 kDa was confirmed by Western blot analysis in 7 out of 14 carcinoma cell lines. Analysis of a large colorectal cancer tissue microarray with 1009 specimens revealed SHIP1 expression in 62% of the samples analyzed. SHIP1 expression was inversely correlated with lymph node metastasis, vascular invasion and tumor grade, and it was positively associated with left-sided tumor localization. Interestingly, a strong relationship between the expression of SHIP1 and nuclear and membranous beta-catenin and the DNA repair genes *MLH1* and *MSH2* was observed.

## 1. Introduction

Colorectal cancer (CRC) is the third most frequently occurring cancer in the western world [1]. A small subgroup (about 15%) of CRCs exhibit inherited or somatic mutations in DNA mismatch repair genes, accompanied by microsatellite instability (MSI) [2]. These cancers are generally different from microsatellite stable CRCs. For instance, they tend to arise in the proximal colon, display enhanced leukocyte infiltration, and their response to chemotherapy is different from that of microsatellite stable (MSS) CRCs [3].

Increasing evidence suggests that inositol 5-phosphatases could play tumor-promoting roles in several carcinomas. The SH2 domain containing inositol 5-phosphatase 2 (SHIP2) has been shown to be associated with poor clinical outcomes in colorectal cancer [4]. Mechanistically, SHIP2 knockdown in breast cancer cells results in reduced metastatic potential [5]. Another inositol 5-phosphatase, synaptojanin 2, has also been shown to be responsible for the metastatic potential of breast cancer cells [6]. These findings prompted us to investigate a putative role of the hematopoietic inositol 5-phosphatase SHIP1 in carcinomas, which shows endogenous expression only in the hematopoietic lineage and therein functions as a negative regulator of hematopoiesis and a suppressor of leukemogenesis [7,8,9,10,11,12,13].

## 2. Experimental Section

### 2.1. Cell Culture

Breast cancer cell line MDA-MB-231 was obtained from American Type Culture Collection (ATCC) (Rockville, MD, USA), and cholangio-carcinoma cell lines EGI-1 and TFK-1 were obtained from the German Collection of Microorganisms and Cell Cultures GmbH (DSMZ), Braunschweig, Germany. Breast cancer cell lines (MDA-MB-231, MDA-MB468, MCF-7), hepatocellular carcinoma cell lines (Hep3B, HepG2 and Huh7), cholangiocarcinoma cell lines (EGI-1, TFK-1, SKCHA-1) and colorectal carcinoma cell lines (SW480, CaCo-2, HCT-116, HT-29 and HROC-24) were cultured in Dulbecco’s Modified Eagle’s Medium (DMEM) medium supplemented with 10% fetal calf serum (FCS), 2 mM L-glutamine and 1 mM sodium pyruvate (Thermo Fisher Scientific, Dreieich, Germany). Jurkat cells were cultured in RPMI medium supplemented with 10% fetal calf serum (FCS), 2 mM L-glutamine and 1 mM sodium pyruvate (Thermo Fisher Scientific, Dreieich, Germany). The TF-1 cells were cultured in RPMI medium supplemented with 10% fetal calf serum (FCS), 2 mM L-glutamine, 1 mM sodium pyruvate (Thermo Fisher Scientific, Dreieich, Germany) and 3 ng/mL recombinant GM-CSF (PeproTech, Hamburg, Germany). All cell lines were cultured at 37 °C with 5% CO_2_ in a humidified atmosphere.

### 2.2. Protein Lysates and Western Blotting

For Western blot analysis, equal amounts of protein determined by the DC Protein Assay (Bio-Rad, Feldkirchen, Germany) were loaded. Western blotting was performed as previously described [14]. Briefly, cells were lysed in NP40 buffer, and the protein concentration was determined as described above. Forty micrograms of total protein was loaded per lane onto a 10% SDS polyacrylamide gel, and the gel was allowed to run until sufficient separation was achieved. Proteins were then transferred to a 0.45 µm nitrocellulose membrane. The membrane was blocked for 1 h at RT in blocking buffer (5% skimmed milk powder in TBS-T), and primary antibody incubation was performed overnight at 12 °C. Primary antibodies were diluted 1:1000 (SHIP1) or 1:3000 (GAPDH) in washing buffer (2.5% skimmed milk powder in TBS-T) and were as follows: anti-SHIP1 (P1C1, sc-8425, Santa Cruz, Dallas, TX, USA) and anti-GAPDH (6C5, sc-32233, Santa Cruz, Dallas, TX, USA). The membranes were then washed three times for 10 min in washing buffer, and secondary antibody incubation was performed for 1 h at RT. Secondary anti-mouse-HRP (Cell Signaling, Cat. 7076) antibody was diluted 1:5000 in washing buffer. Membranes were washed four times for 15 min in TBS-T and twice for 10 min in TBS. Specific protein signals were detected by chemiluminescence.

### 2.3. Multi-Tumor Tissue Microarray

A multi-tumor tissue micro-array (TMA), including 10–60 (total: 2089) samples, each from 72 different human tumor types and subtypes, made from formalin-fixed paraffin-embedded tissue samples, was used to analyze SHIP-1 expression in this study. The exact composition of the TMA is given in the supplementary information. For the TMA set, tissue cylinders with a diameter of 0.6 mm were punched from tumor areas of each tissue block and brought into a recipient paraffin block using the paraffin sectioning aid system (adhesive-coated slides (PSA-CS4x), adhesive tape, and UV lamp; Instrumedics, Inc., Hackensack, NJ, USA). All tumor samples represented in these TMAs were obtained from the archives of the Institute of Pathology of the University Medical Center Hamburg Eppendorf. The use of archived diagnostic leftover tissues for the manufacturing of tissue microarrays and their analysis for research purposes, as well as patient data analysis, has been approved by local laws (HmbKHG, §12,1) and by the local ethics committee (Ethics Commission Hamburg, WF-049/09 and PV3652). All work was carried out in compliance with the Helsinki Declaration.

### 2.4. Immunohistochemistry

Heat-induced autoclave antigen retrieval was performed on deparaffinized sections at 120 °C for 5 min in Tris-EDTA-Citrate (TEC) buffer (pH 7.8), followed by peroxidase blocking (3% H_2_O_2_ in methanol) for 10 min (21 °C, pH 7.6). Sections were incubated with the pre-diluted primary antibody SHIP-1 (monoclonal rabbit, clone P1C1, dilution 1:50, Santa Cruz, Dallas, TX, USA, cat#sc-8425). Visualization of the immunoreaction was performed using DAB-chromogen/EnVision Polymer-HPR (K3468 and K4001; DAKO North America, Carpinteria, CA, USA). Mild counterstaining was performed with hematoxylin for 30 s. For tumor samples, the staining intensity was scored using a four-step scale (0, 1+, 2+, or 3+). In addition, the percentage of positive cells was estimated. The results of all TMAs were then grouped into three categories: negative (no detectable staining), weak (1+ staining in ≤70% of tumor cells or 2+ staining in ≤30% of tumor cells), moderate (1+ staining in >70% of tumor cells or 2+ staining in >30% but ≤70% of tumor cells or 3+ staining in ≤30% of tumor cells and strong (2+ staining in >70% or 3+ staining in >30% of tumor cells). The performance of statistical analysis, as well as IHC staining of MLH1, MSH2 and β-Catenin, is described elsewhere [15].

### 2.5. Bioinformatical Analysis of TCGA Data

Data from the Cancer Genome Atlas project were downloaded either directly from TCGA or from cbioportal.org. To normalize SHIP1 mRNA expression to leukocyte marker expression, z scores of SHIP1 and leukocyte markers ITGB2, ITK, BTK, LST1, PTPRC, CD4 and CD48 were calculated based on the analyzed colorectal cancer samples. The mean z score of the seven leukocyte markers used was subtracted from the z scored SHIP1 expression in the corresponding sample. Statistical analysis of leukocyte normalized SHIP1 expression with different clinicopathological parameters was carried out using one-way ANOVA. To generate the heatmap, the environment of R studio was used. Statistical analysis of miR-155 expression between low and high SHIP1/leukocyte score was done by student’s t-test, statistical analysis of mutation statuses and clinicopathological parameters performed using Fisher’s exact test [15].

### 2.6. Phosphatase Assay

The activity of SHIP1-expressing cell lines was assessed as described previously [7]. Briefly, cells were seeded at a density of 4 × 10^5^/10 cm dish and cultured for 4 days in DMEM, 10% FCS, and 1% p/s. Cells were lysed by NP40-lysis, and SHIP1 was isolated out of the cell lysate by immunoprecipitation using an anti-SHIP1 P1C1 mouse monoclonal antibody immobilized to Protein G Sepharose beads. The phosphatase activity of SHIP1 was measured by a malachite-green based phosphatase assay using ci8-PtdIns(3,4,5)P_3_ as a substrate. To calculate the specific activity, the concentration of SHIP1 was determined by Western blot analysis using recombinant SHIP1 as a standard. Data are presented as the means of three replicates.

## 3. Results

### 3.1. SHIP1 Expression in Human Carcinoma Cell Lines and Clinical Samples

**Figure 1:**
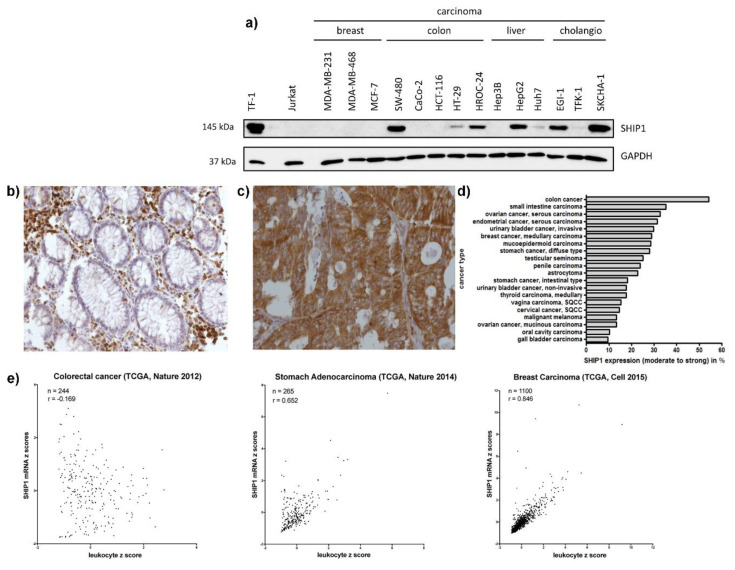
**Expression of SHIP1 in carcinomas and sarcomas.** (**a**) Lysates from indicated cell lines were electrophoretically separated using SDS-PAGE, and a Western blot was performed with monoclonal antibodies against SHIP1 and GAPDH. (**b**,**c**). Human tissue samples spotted on a multi-tumor tissue microarray were stained for SHIP1 via immunohistochemistry. Representative images of SHIP1 immunohistochemistry in colon tissues taken at 100× magnification are shown. Positive SHIP1 staining in lymphocytes of normal colon tissue was observed, while epithelial cells were negative (**b**). Strong SHIP1 staining was detected in colon cancer cells (**c**). (**d**) Twenty cancer types and subtypes with the highest frequency of moderate to strong SHIP1 expression are shown. Only cancers with at least ten specimens were included. (**e**) Publicly available RNA Seq expression data from The Cancer Genome Atlas (TCGA) was used to analyze SHIP1 expression on the RNA level. SHIP1 mRNA expression from a colorectal cancer, stomach adenocarcinoma and breast carcinoma dataset was correlated with a set of leukocyte markers (see Section 2.5), showing that SHIP1 mRNA expression is independent of leukocyte marker expression in colorectal cancer but not in breast carcinoma.

To analyze the expression of the hematopoietic inositol 5-phosphatase SHIP1 in solid human cancers, we first performed Western blot analysis of carcinoma cell lines derived from breast cancer (MDA-MB-231, MDA-MB-468, MCF-7), colorectal cancer (SW-480, CaCo2, HT-29, HCT-116, HROC-24), liver cancer (Hep3B, HepG2, Huh7) and cholangio carcinoma (EGI-1, TFK-1, SKCHA-1). The leukemia cell line TF-1 was used as a positive control, and the T-ALL cell line Jurkat, which does not express SHIP1 proteins, was used as a negative control. Of the 14 carcinoma cell lines analyzed, seven showed detectable amounts of SHIP1 at the expected molecular mass of 145 kDa (Figure 1a). We then performed a SHIP1 phosphatase assay based on the malachite-green dependent detection of phosphate liberated in the course of the SHIP1 catalyzed dephosphorylation of PtdIns(3,4,5)P_3_ using four SHIP1 expressing model carcinoma cell lines (HT-29, SW-480, SKCHA-1 and HepG2). SHIP1 activity was detectable in all cell lines tested; however, specific activities varied between individual cell lines and were significantly lower in HT-29 cells (Figure S1). Next, a multi-tumor tissue microarray was assayed to validate these results with primary tumor samples. The specific staining of SHIP1 on formalin-fixed paraffin-embedded cells was demonstrated by using a doxycycline-inducible SHIP1 expression system (Figure S2) as described previously [9]. In addition, we performed control staining with isotype control antibodies on the tumor tissue microarrays, which did not show unspecific staining of cells (Figure S3). Several tumor entities derived from epithelial or mesenchymal tissues showed high percentages of moderate to strong SHIP1 protein expression. In 48 out of the 72 cancer entities analyzed, moderate or strong expression of SHIP1 was detected, and 26 out of the 48 positive entities showed a staining frequency of at least 10% (Figure 1d, Table S1).

The highest frequency of expression was observed in colon cancer, wherein 19 out of 35 (54%) specimens showed moderate to strong expression of SHIP1. Healthy colon mucosa only showed expression in infiltrating leukocytes. Interestingly, other gastrointestinal-derived carcinomas also showed high percentages of SHIP1 expression (Figure 1b–d).

Using publicly available RNA sequencing data from The Cancer Genome Atlas project (TCGA), we further investigated the expression of SHIP1 on the level of RNA. Because SHIP1 is endogenously expressed at very high levels in leukocytes, correlation analyses between SHIP1 expression and leukocyte markers were performed. Only limited variation was observed between different leukocyte markers (Figure S4). Intriguingly, in clear contrast to breast cancer, where SHIP1 and leukocyte markers were greatly correlated (r = 0.846), in colorectal cancer, SHIP1 mRNA was not correlated with leukocyte markers (r = −0.169) (Figure 1e). This indicates that SHIP1 mRNA expression in colorectal cancer is not a footprint of leukocyte infiltration and is upregulated in the cancer cells themselves.

### 3.2. Correlation between SHIP1 Expression and Clinicopathological Parameters

**Figure 2:**
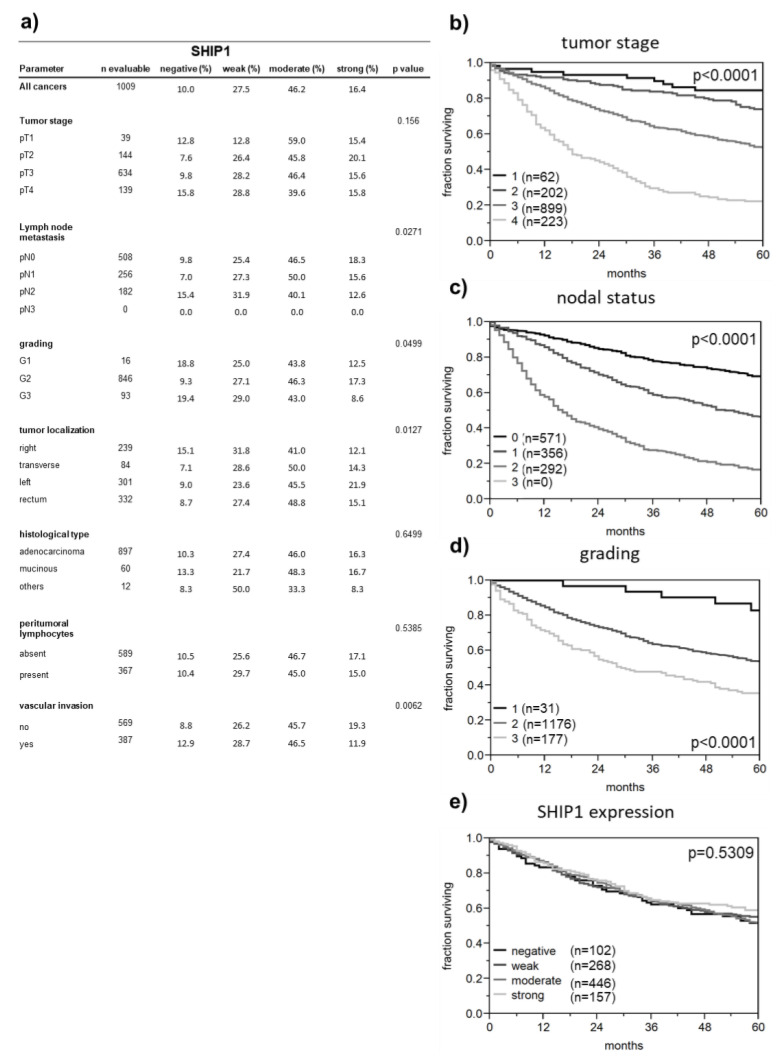
Association of SHIP1 expression with clinicopathological parameters in colorectal cancer. (**a**) Relationship between SHIP1 staining, tumor phenotype and clinical parameters. Patient survival is related to tumor stage (**b**), nodal status (**c**), and tumor grade (**d**), but not to SHIP1 expression (**e**).

The high and abundant SHIP1 expression in colorectal cancer tempted us to investigate the putative role of SHIP1 using a colorectal cancer tissue microarray, including follow-up data. Of the 1009 analyzable specimens, only 10% did not show protein expression of SHIP1, and 62.6% expressed SHIP1 at moderate to strong levels. Expression of SHIP1 was inversely associated with lymph node metastasis, tumor grading and vascular invasion (Figure 2a); however, no correlation was observed between SHIP1 expression and patient survival (Figure 2e). The integrity of the data set is demonstrated as a strong association between patient survival and the common prognostic marker tumor stage, nodal status and tumor grade (Figure 2b–d).

**Figure 3:**
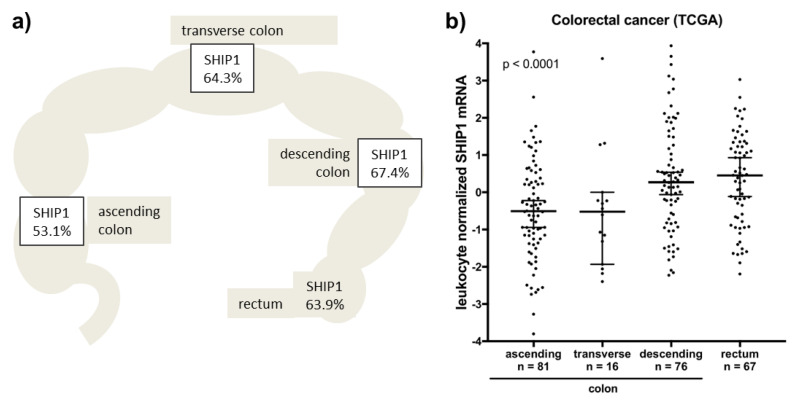
SHIP1 expression is correlated with left-sided colorectal cancer. (**a**) Moderate and strong SHIP1 protein expression (Figure 2a) is significantly related to left-sided tumor localization (*p* = 0.0127). (**b**) SHIP1 mRNA expression (from TCGA) was normalized to leukocyte markers and analyzed for association with colorectal cancer localization (*p* < 0.0001).

In addition, SHIP1 was associated with left-sided tumor localization (Figure 2a and Figure 3a). This finding was further validated at the RNA level using TCGA data. Although SHIP1 mRNA was not correlated with leukocyte markers in colorectal cancer, it is still obvious that increased leukocyte infiltration will result in enhanced SHIP1 expression in tumor tissue. Therefore, we used subtracted z scores of SHIP1 mRNA and leukocyte markers to compensate for different levels of leukocytes in the tumor tissue. This compensation resulted in a pattern of SHIP1 mRNA in tumor localization, which was very similar to our observed protein expression (Figure 3b).

### 3.3. Downregulation of SHIP1 in Microsatellite Unstable Colorectal Cancers

**Figure 4:**
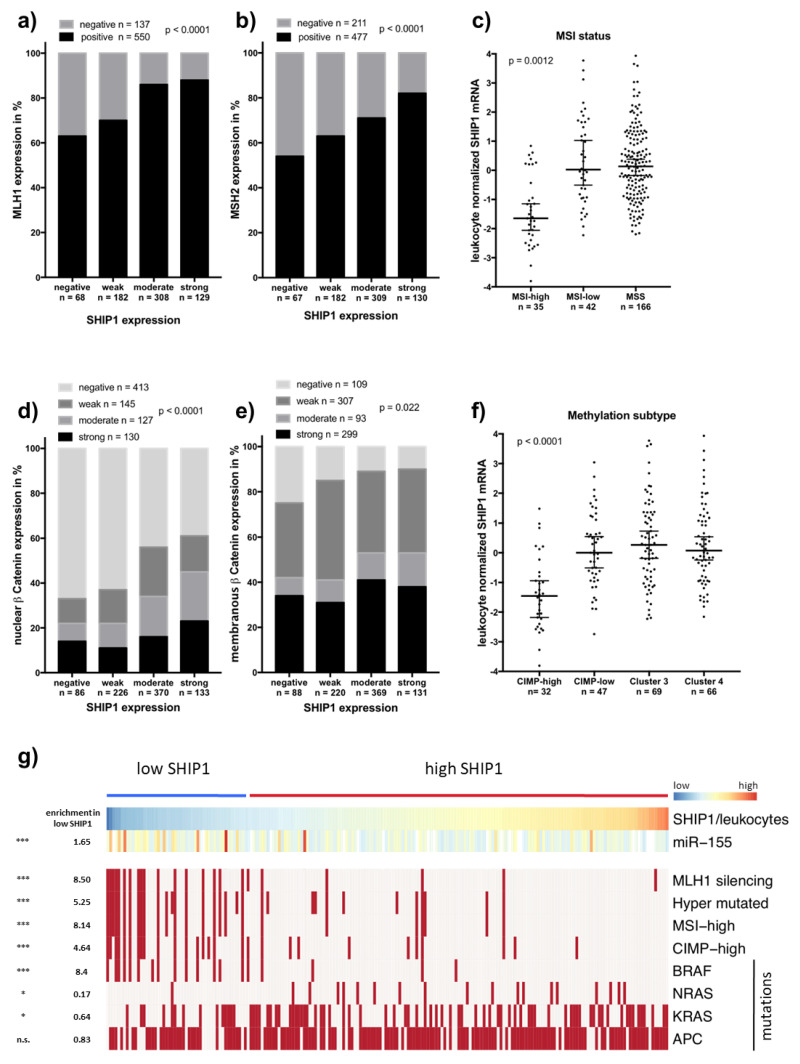
The absence of SHIP1 expression in colorectal cancer is linked to microsatellite instability. (**a**,**b**) Immunohistochemistry-scored SHIP1 expression was correlated with expression of MLH1 and MSH2 as markers for microsatellite instability (*p* > 0.0001, each). (**c**) Leukocyte normalized SHIP1 mRNA expression from TCGA was analyzed for association with microsatellite status (*p* = 0.0012). (**d**,**e**) Immunohistochemistry-scored SHIP1 expression was correlated with the expression of nuclear and membranous β-Catenin (*p* > 0.0001 and *p* = 0.0022, respectively). (**f**) Leukocyte normalized SHIP1 mRNA expression was analyzed for association with methylation subtypes (*p* < 0.0001). (**g**) Leukocyte normalized SHIP1 mRNA expression was classified into low SHIP1 expression (low quartile) and high (upper three quartiles), and hierarchical clustering was performed, showing enrichment of microsatellite instability markers.

Protein expression of SHIP1 was further correlated with molecular markers published previously. Interestingly, SHIP1 was strongly associated with the DNA mismatch repair proteins MLH1 and MSH2 (Figure 4a,b), of which their loss of expression results in microsatellite instability. The loss of SHIP1 expression in microsatellite unstable colorectal carcinomas was independently validated using TCGA data (Figure 4c) wherein SHIP1 mRNA expression was greatly decreased in tumors with high levels of microsatellite instability (MSI-high). Nuclear expression of β-Catenin, which indicates activated WNT signaling, was also strongly associated with SHIP1 expression (Figure 4d), as well as was expression of membranous β-Catenin (Figure 4e). Finally, grouping of SHIP1 mRNA relative to leukocyte markers into a low (first quartile) and a high expression group was able to enrich several markers of microsatellite instability, including MLH1 silencing, hypermutations, high levels of CpG-island methylation phenotype and *BRAF* mutations (Figure 4f,g). Interestingly, in this group, decreased rates of *NRAS* and *KRAS* mutations were observed. Additionally, microRNA-155, of which SHIP1 is a known target, is enhanced in the low SHIP1 expression group and is commonly upregulated in microsatellite unstable colorectal cancers.

## 4. Discussion

Here, we report that SHIP1 is frequently expressed in human carcinomas and sarcomas. In 48 out of the 72 cancer entities analyzed, moderate or strong expression of SHIP1 was detected, and 26 out of the 48 positive entities showed staining of SHIP1 in at least 10% of the samples analyzed. Previous research on SHIP1 has concentrated on the effects of SHIP1 in hematopoietic cells, where SHIP1 is endogenously expressed. In these cells, SHIP1 has been shown to function as a tumor suppressor in acute myeloid leukemia due to the reduction of PI3K-AKT signaling [7,8,9,10,11,12,13]. This role does not predict an upregulation of SHIP1 expression in solid tumors, which, however, is the case. Our data demonstrate that SHIP1 expression in colorectal cancer where the strongest upregulation was observed is not directly associated with patient survival but is inversely correlated with lymph node metastasis, tumor grade and vascular invasion. Also, SHIP1 was downregulated in microsatellite unstable colorectal cancers assayed by the absence of MLH1 or MSH2 expression. In addition, SHIP1 was correlated with nuclear β-catenin staining, which is in line with the observation that WNT/β-catenin signaling is downregulated in microsatellite unstable colorectal cancers [16]. These results were independently validated using publicly available RNA sequencing data from TCGA. However, it should be noted that leukocyte infiltration is a common characteristic of MSI in colorectal cancer, which obviously results in the upregulation of leukocyte markers in MSI CRC samples. Therefore, our used compensation method to predict SHIP1 mRNA expression in CRC samples is necessary. On the other hand, the observed enrichment of MSI markers in the low SHIP1 group could be partly due to the leukocyte infiltration of MSI CRCs. However, without that compensation, these patterns were also observed, but with a lower extent (data not shown).

So far, the mechanism(s) by which SHIP1 is upregulated in several solid tumors, especially in colorectal cancer, is speculative, but common mechanisms could include aberrant DNA demethylation of the *INPP5D* promotor and/or upregulation or downregulation of a transcriptional activator or repressor.

The cause by which SHIP1 is especially downregulated in microsatellite unstable colorectal cancers is also as much unknown as its upregulation in colorectal cancer in general. One tempting explanation could be the observed upregulation of miR-155 in the SHIP1 mRNA low group. In fact, miR-155 is upregulated in MSI CRCs and is known as a direct down regulator of SHIP1 in hematopoietic cells [17].

If SHIP1 has a functional role in colorectal cancer, it could be involved in PI3K/AKT/GSK3β/β-catenin signaling. SHIP1 dephosphorylates PtdIns(3,4,5)P_3_ at the D5 position and probably leads to increased PtdIns(3,4)P_2_ amounts which may trigger oncogenic signaling [18,19]. Regarding this, the finding of catalytically active SHIP1 in four model carcinoma cell lines shown here for the first time is of special note. As demonstrated, SHIP1 specific activity varied between those cell lines and was significantly lower in HT-29 cells. This might be a result of cell line-specific SHIP1 mutations leading to reduced catalytic activity. According to the COSMIC database, various SHIP1 mutations are listed for carcinoma samples, i.e., 5.1% of 2314 listed colorectal carcinoma samples were found to be mutated (date of access 08.06.2020). Furthermore, in a previous publication, we found reduced activity of patient-derived SHIP1 mutations derived from leukemia samples [7].

In summary, hematopoietic tumor suppressor SHIP1 is frequently expressed in human carcinomas and sarcomas. The abundant and high expression of SHIP1 in colorectal cancer was observed at the RNA and protein levels. While SHIP1 was not correlated with patient survival, it was strongly downregulated at both RNA and protein levels in microsatellite unstable colorectal cancers. This probably unravels a new molecular mechanism between microsatellite instability and SHIP1 expression and offers the possibility that SHIP1 could be used as a marker to predict microsatellite instability in colorectal cancer.

## Supplementary Materials

The following are available online at https://www.mdpi.com/2227-9059/8/7/215/s1, Table S1: Expression of SHIP1 in solid human tumors. Supplementary Figure S1: Phosphatase assay of carcinoma cell lines ectopically expressing SHIP1. Supplementary Figure S2: Validation of specificity of the anti-SHIP1 antibody. Supplementary Figure S3: Validation of specificity of the immune histochemistry staining on tissue microarrays using anti-SHIP1 antibody. Figure S4: Standard error of the leukocyte z score.

## References

[1] Ferlay J., Soerjomataram I., Dikshit R., Eser S., Mathers C., Rebelo M., Parkin N.M., Forman D., Bray F. (2015). Cancer incidence and mortality worldwide: Sources, methods and major patterns in GLOBOCAN 2012. Int. J. Cancer.

[2] Sinicrope F.A., Sargent D.J. (2012). Molecular Pathways: Microsatellite Instability in Colorectal Cancer: Prognostic, Predictive, and Therapeutic Implications. Clin. Cancer Res..

[3] Boland C.R., Goel A. (2010). Microsatellite Instability in Colorectal Cancer. Gastroenterology.

[4] Yang J., Fu M., Ding Y., Weng Y., Fan W., Pu X., Ge Z., Zhan F., Ni H., Zhang W. (2014). High SHIP2 expression indicates poor survival in colorectal cancer. Dis. Markers.

[5] Sharma V.P., Eddy R., Entenberg D., Kai M., Gertler F.B., Condeelis J. (2013). Tks5 and SHIP2 regulate invadopodium maturation, but not initiation, in breast carcinoma cells. Curr. Biol..

[6] Ben-Chetrit N., Chetrit D., Russell R., Körner C., Mancini M., Abdul-Hai A., Itkin T., Carvalho S., Cohen-Dvashi H., Koestler W.J. (2015). Synaptojanin 2 is a druggable mediator of metastasis and the gene is overexpressed and amplified in breast cancer. Sci. Signal..

[7] Brauer H., Strauss J., Wegner W., Müller-Tidow C., Horstmann M., Jücker M. (2012). Leukemia-associated mutations in SHIP1 inhibit its enzymatic activity, interaction with the GM-CSF receptor and Grb2, and its ability to inactivate PI3K/AKT signaling. Cell. Signal..

[8] Gilby D.C., Goodeve A.C., Winship P.R., Valk P.J., Delwel R., Reilly J.T. (2007). Gene structure, expression profiling and mutation analysis of the tumour suppressor SHIP1 in Caucasian acute myeloid leukaemia. Leukemia.

[9] Horn S., Endl E., Fehse B., Weck M.M., Mayr G.W., Jücker M. (2004). Restoration of SHIP activity in a human leukemia cell line downregulates constitutively activated phosphatidylinositol 3-kinase/Akt/GSK-3β signaling and leads to an increased transit time through the G1 phase of the cell cycle. Leukemia.

[10] Liu Q., Oliveira-Dos-Santos A.J., Mariathasan S., Bouchard D., Jones J., Sarao R., Kozieradzki I., Ohashi P.S., Penninger J.M., Dumont D.J. (1998). The inositol polyphosphate 5-phosphatase ship is a crucial negative regulator of B cell antigen receptor signaling. J. Exp. Med..

[11] Luo J.-M., Yoshida H., Komura S., Ohishi N., Pan L., Shigeno K., Hanamura I., Miura K., Iida S., Ueda R. (2003). Possible dominant-negative mutation of the SHIP gene in acute myeloid leukemia. Leukemia.

[12] Luo J.-M., Liu Z.-L., Hao H.-L., Wang F.-X., Dong Z.-R., Ohno R. (2004). Mutation analysis of SHIP gene in acute leukemia. Zhongguo Shi Yan Xue Ye Xue Za Zhi.

[13] Täger M., Horn S., Latuske E., Ehm P., Schaks M., Nalaskowski M., Fehse B., Fiedler W., Stocking C., Wellbrock J. (2017). SHIP1, but not an AML-derived SHIP1 mutant, suppresses myeloid leukemia growth in a xenotransplantation mouse model. Gene Ther..

[14] Grabinski N., Bartkowiak K., Grupp K., Brandt B., Pantel K., Jücker M. (2011). Distinct functional roles of Akt isoforms for proliferation, survival, migration and EGF-mediated signalling in lung cancer derived disseminated tumor cells. Cell. Signal..

[15] Tennstedt P., Fresow R., Simon R., Marx A.H., Terracciano L., Petersen C., Sauter G., Dikomey E., Borgmann K. (2013). RAD51 overexpression is a negative prognostic marker for colorectal adenocarcinoma. Int. J. Cancer.

[16] Panarelli N.C., Vaughn C.P., Samowitz W.S., Yantiss R.K. (2015). Sporadic Microsatellite Instability-High Colon Cancers Rarely Display Immunohistochemical Evidence of Wnt Signaling Activation. Am. J. Surg. Pathol..

[17] O’Connell R.M., Chaudhuri A.A., Rao D.S., Baltimore D. (2009). Inositol phosphatase SHIP1 is a primary target of miR-155. Proc. Natl. Acad. Sci. USA.

[18] Franke T.F., Kaplan D.R., Cantley L.C., Toker A. (1997). Direct regulation of the Akt proto-oncogene product by phosphatidylinositol-3,4-bisphosphate. Science.

[19] Scheid M.P., Huber M., Damen J.E., Hughes M., Kang V., Neilsen P., Prestwich G.D., Krystal G., Duronio V. (2002). Phosphatidylinositol (3,4,5)P _3_ Is Essential but Not Sufficient for Protein Kinase B (PKB) Activation; Phosphatidylinositol (3,4)P _2_ Is Required for PKB Phosphorylation at Ser-473. J. Biol. Chem..

