# Supplementary material for: Ectopic Expression of Hematopoietic SHIP1 in Human Colorectal Cancer
Source: Biomedicines. 2020 Jul 15;8(7):215. doi: 10.3390/biomedicines8070215 (PMC7400281; doi:10.3390/biomedicines8070215)
Supplement: Supplementary file 1 [file biomedicines-08-00215-s001.pdf]

Supporting information

Supplementary Table 1: Expression of SHIP1 in human solid tumors. Tumor samples on a tissue microarray were stained against SHIP1 and scored into negative, weak, moderate or strong as described in the material and methods section. Only tumor entities with at least ten analyzable specimens are shown.

	Tumor type	Immunostaining of SHIP1 in human solid tumor samples								
		n analyzable	negative (n)	weak (n)	moderate (n)	strong (n)	negative (%)	weak (%)	moderate (%)	strong (%)
**skin tumors**	basal cell adenoma	16	12	4	0	0	75	25	0	0
	basalioma	23	16	6	1	0	70	26	4	0
	malignant melanoma	15	7	6	1	1	47	40	7	7
	pilomatrixoma	21	5	13	3	0	24	62	14	0
	skin cancer, SQCC	27	21	4	2	0	78	15	7	0
**respiratory tract tumors**	larynx, carcinoma	32	24	6	2	0	75	19	6	0
	lung cancer, adenocarcinoma	45	27	15	3	0	60	33	7	0
	lung cancer, large cell cancer	23	13	9	1	0	57	39	4	0
	lung cancer, SQCC	34	25	9	0	0	74	26	0	0
	mucoepidermoid carcinoma	14	6	4	3	1	43	29	21	7
	oral cavity, carcinoma	29	22	4	2	1	76	14	7	3
	parotis, pleomorphic adenoma	41	33	8	0	0	80	20	0	0
	Warthin's tumor	37	13	24	0	0	35	65	0	0
**gynecological tumors**	breast cancer, apocrine carcinoma	11	8	3	0	0	73	27	0	0
	breast cancer, ductal carcinoma	36	32	4	0	0	89	11	0	0
	breast cancer, kribriform carcinoma	15	15	0	0	0	100	0	0	0
	breast cancer, lobulary carcinoma	34	29	5	0	0	85	15	0	0
	breast cancer, medullary carcinoma	45	10	22	12	1	22	49	27	2
	breast cancer, mucinous carcinoma	39	32	6	1	0	82	15	3	0
	breast cancer, phylloid carcinoma	15	11	4	0	0	73	27	0	0
	breast cancer, tubulary carcinoma	20	16	4	0	0	80	20	0	0
	cervical cancer, adenocarcinoma	26	10	14	2	0	38	54	8	0
	cervical cancer, SQCC	48	2	39	5	2	4	81	10	4
	endometrial cancer, endometroid carcinoma	52	26	23	3	0	50	44	6	0
	endometrial cancer, serous carcinoma	35	13	11	10	1	37	31	29	3
	ovarian cancer, Brenner tumor	21	7	14	0	0	33	67	0	0
	ovarian cancer, endometroid carcinoma	19	8	10	1	0	42	53	5	0
	ovarian cancer, mucinous carcinoma	30	16	10	4	0	53	33	13	0
	ovarian cancer, serous carcinoma	52	11	24	17	0	21	46	33	0
	teratoma	14	8	4	2	0	57	29	14	0
	vagina carcinoma, SQCC	13	6	5	2	0	46	38	15	0
	vulva carcinoma, SQCC	38	28	8	2	0	74	21	5	0
**gastrointestinal tumors**	colon adenoma, low grade	11	1	6	3	1	9	55	27	9
	colon adenoma, high grade	14	1	4	6	3	7	29	43	21
	colon cancer	35	9	7	13	6	26	20	37	17
	esophageal carcinoma, adenocarcinoma	46	27	15	2	2	59	33	4	4
	esophageal carcinoma, SQCC	40	30	8	2	0	75	20	5	0
	gall bladder carcinoma	21	14	5	2	0	67	24	10	0
	gastrointestinal stroma tumor (GIST)	31	28	2	1	0	90	6	3	0
	hepatocellular carcinoma	46	41	5	0	0	89	11	0	0
	pancreatic cancer, ductal adenocarcinoma	31	20	11	0	0	65	35	0	0
	pancreatic cancer, papilla, adeno	13	6	6	1	0	46	46	8	0
	small intestine carcinoma	17	7	4	3	3	41	24	18	18
	stomach cancer, diffuse type	25	8	10	3	4	32	40	12	16
	stomach cancer, intestinal type	33	12	15	4	2	36	45	12	6
	oncocytoma	43	42	1	0	0	98	2	0	0
**genitourinary tract tumors**	testis, non-seminoma	14	10	3	1	0	71	21	7	0
	testis, seminoma	60	33	12	10	5	55	20	17	8
	penile carcinoma	21	9	7	5	0	43	33	24	0
	prostate cancer	45	43	1	1	0	96	2	2	0
	renal cell cancer, chromophobic	36	35	1	0	0	97	3	0	0
	renal cell cancer, clear cell	44	27	14	2	1	61	32	5	2
	renal cell cancer, papillary	18	11	7	0	0	61	39	0	0
	urinary bladder cancer, invasive (pT2-4)	47	13	20	14	0	28	43	30	0
	urinary bladder cancer, non-invasive (pTa)	51	13	29	9	0	25	57	18	0
**neuroendocrine tumors**	adrenal cortex, adenoma	12	6	6	0	0	50	50	0	0
	carcinoid	19	14	4	1	0	74	21	5	0
	paraganglioma	24	16	7	1	0	67	29	4	0
	phaeochromocytoma	55	35	19	1	0	64	35	2	0
	thyroid carcinoma, follicular	39	38	1	0	0	97	3	0	0
	thyroid carcinoma, medullary	17	11	3	3	0	65	18	18	0
	thyroid carcinoma, papillary	36	31	4	1	0	86	11	3	0
	thyroid, adenoma	43	41	2	0	0	95	5	0	0
**neuronal tumors**	astrocytoma	22	11	6	5	0	50	27	23	0
	oligodendroglioma	17	14	2	1	0	82	12	6	0
	neuroblastoma	35	27	8	0	0	77	23	0	0
**soft tissue tumors**	carcinosarcoma	25	14	11	0	0	56	44	0	0
	giant cell tumor of the tendon sheat	18	5	10	3	0	28	56	17	0
	leiomyosarcoma	22	15	7	0	0	68	32	0	0
	malignant fibrous histiocytoma	17	4	8	5	0	24	47	29	0
	malignant schwannoma	11	8	3	0	0	73	27	0	0
	neurofibroma	15	11	4	0	0	73	27	0	0

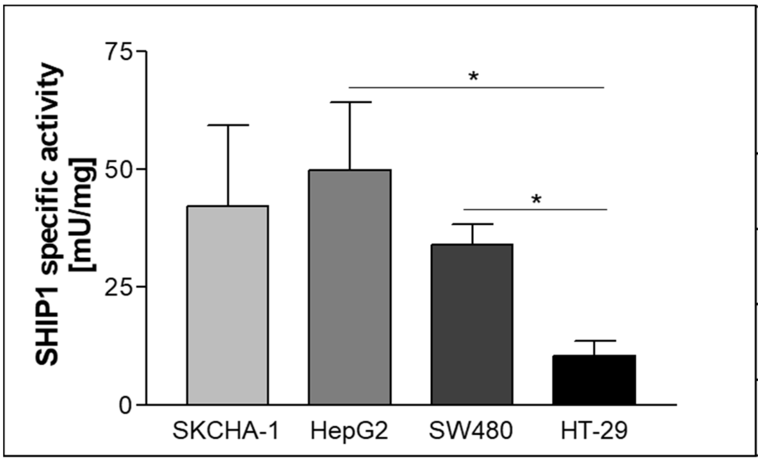

	Specific activity (mU/mg)
SKCHA-1	42.18 ± 29.71
HepG2	49.88 ± 20.16
SW480	33.94 ± 6.24
HT-29	10.55 ± 5.33

Supplementary Figure 1: Phosphatase assay of carcinoma cell lines ectopically expressing SHIP1. The enzymatic activity of SHIP1 expressed in selected carcinoma cell lines was assessed by a malachite green based inositol phosphatase assay using a shortened derivate of PtdIns(3,4,5)P_3 (ci8-PtdIns(3,4,5)P_3) as a substrate. SHIP1 was isolated out of whole cell lysates by immunoprecipitation using SHIP1 specific antibody and the amount of SHIP1 in the assay was determined by Western Blot analysis using recombinant SHIP1 as a standard in order to calculate the specific activity. All measurements were done in threefold determination. Significances were calculated by unpaired Students t-test.

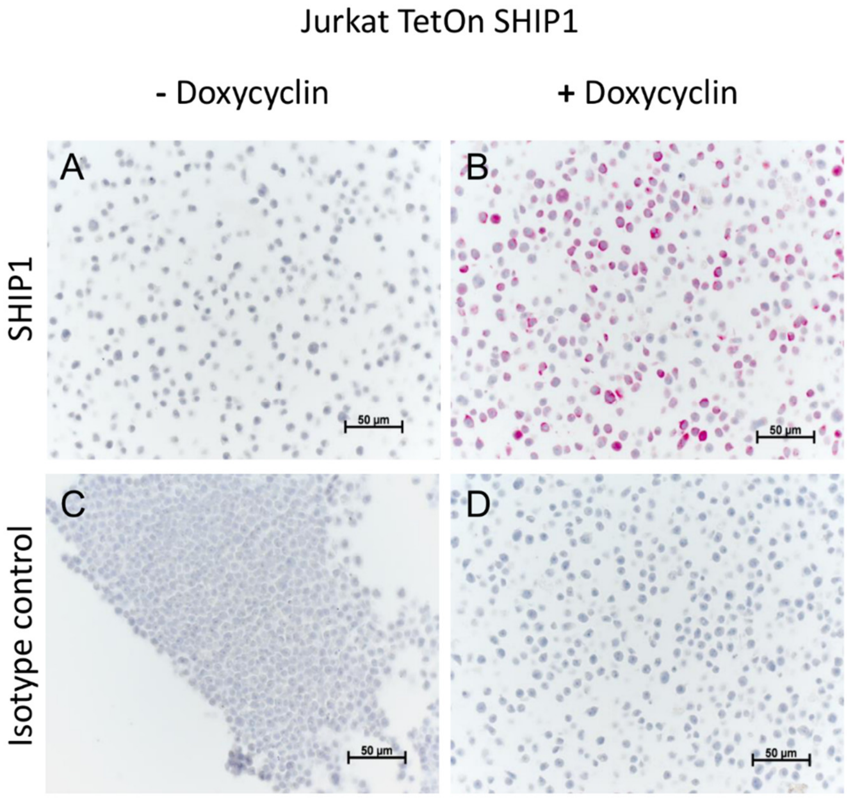

Supplementary Figure 2: Validation of specificity of the anti-SHIP1 antibody. Jurkat-TetOn cells, transduced with SHIP1 under the control of a doxycycline inducible promotor were either treated with doxycycline (1µg/ml) or an equal volume of ethanol. Cells were stained either with the anti-SHIP1 antibody (**a), b)**) or with an isotype control antibody (**c), d)**).

a)

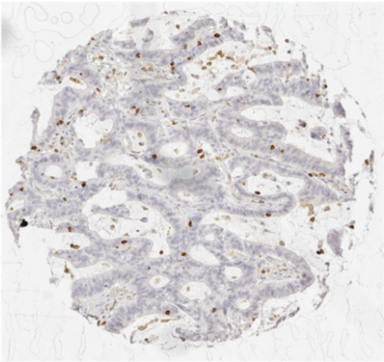

b)

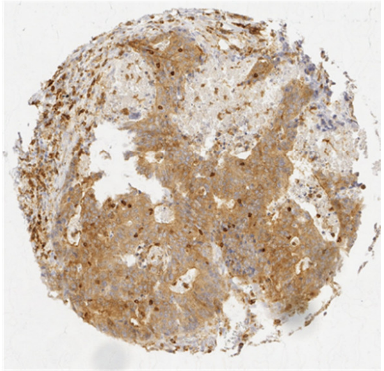

c)

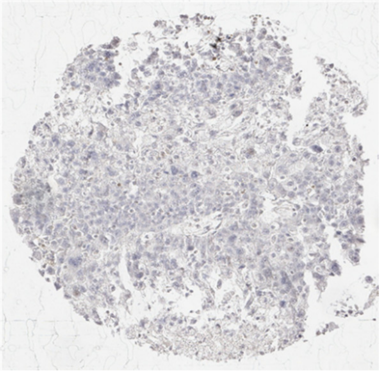

Supplementary Figure 3: Validation of specificity of the immune histochemistry staining on tissue microarrays using anti-SHIP1 antibody. a) A tissue microarray with colon tissue of a healthy person was stained with anti-SHIP1 antibody. Colon cells are negative, whereas immune cells show a strong SHIP-1 staining. b) A tissue microarray with colon cancer was stained with anti-SHIP1 antibody. SHIP-1 positive colon cancer cells and immune cells are detectable. c) Control staining of a tissue microarray with colon cancer was performed with an isotype control antibody. Neither cancer cells nor immune cells show detectable staining.

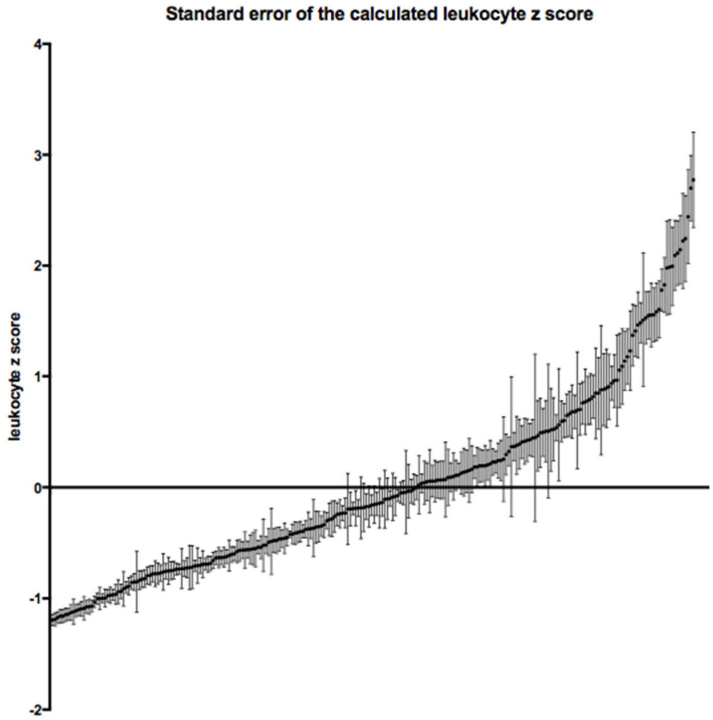

Supplementary Figure 4: Standard error of the leukocyte z score. For the colorectal cancer TCGA data, z score of leukocyte markers used (ITGB2, ITK, BTK, LST1, PTPRC, CD4 and CD48) was calculated and their level of concurrence was expressed by calculating the standard error of leukocyte markers for each sample, showing limited variability.
